# Tooth loss trajectories and their association with all-cause mortality among older Chinese adults

**DOI:** 10.3389/froh.2025.1535708

**Published:** 2025-02-26

**Authors:** Xiaoming Zhang, Rui Zeng, Dongmei Ye, Mengxia Shi, Aizhang Zhu, Lihuan Chen, Tenghui Fan, Ke Zhu, Fayi Xie, Wan Zhu, Yufei Zeng, Jiang Wang, Wenwu Zhang

**Affiliations:** ^1^ Department of Emergency, The People’s Hospital of Baoan Shenzhen, Shenzhen, China; ^2^ School of Clinical Medicine, Jinggangshan University, Ji'an, Jiangxi, China; ^3^ School of Basic Medicine, Jinggangshan University, Ji'an, Jiangxi, China; ^4^ Online Collaborative Research Center for Evidence-Based Medicine Ministry of Education, Jinggangshan University Branch, Ji'an, Jiangxi, China; ^5^ School of Chinese Medicine, Jinggangshan University, Ji'an, Jiangxi, China

**Keywords:** all-cause mortality, tooth loss trajectories, older Chinese adults, Growth mixture models (GMM), CLHLS

## Abstract

**Background:**

The association between tooth loss trajectories and all-cause mortality has not been sufficiently explored. This study aims to examine the relationship between tooth loss trajectories and all-cause mortality in Chinese adults aged 65 years and older.

**Methods:**

This study included 3,726 participants from the Chinese Longitudinal Healthy Longevity Study (CLHLS). The inclusion criteria required participants to be aged 65 years or older, with complete data on tooth count at baseline and at least one follow-up survey. Participants were excluded if they had missing data on death, time to death, or if their they reported tooth count showed an abnormally high increase. The mean age of participants was 85.16 ± 10.7 years. To identify distinct trajectories of tooth loss, growth mixture models (GMM) were employed. Cox regression analysis was utilized to assess the association between tooth loss trajectories and all-cause mortality. Sensitivity analyses were conducted to test the robustness of the findings, while subgroup analyses were performed to explored potential variations in association across different demographic groups.

**Results:**

The prevalence of edentulism at baseline was 37.13%, with a cumulative incidence of 15.8% over 10-year period. Three distinct tooth loss trajectories were identified during follow-up of 9.41 years: (1) progressively mild loss: comprising 312 participants (8.37%); (2) progressively severe loss, comprising 505 participants (13.55%); and (3) edentulism group, comprising 2,909 participants (78.07%). The median follow-up times for each group were 5.91 years, 3.44 years, and 1.84 years, respectively. During the follow-up period, the number of deaths were 114 (36.54%) in the progressively mild loss group, 274 (54.26%) in the progressively severe loss group, and 2,284 (78.51%) in the edentulism group. Compared to the progressively mild loss group, the hazard ratio (HR) for all-cause mortality was 1.29 (95% CI, 1.01–1.64) in the progressively severe loss group, and 1.60 (95% CI, 1.28–1.99) in the edentulism group.

**Conclusions:**

This study identified three distinct tooth loss trajectories among older Chinese adults, with the edentulism group exhibiting the strongest association with all-cause mortality. These findings highlight the crucial importance of maintaining oral health and preserving natural teeth to promote longevity and improve overall health outcomes in older adults.

## 1 Introduction

The global aging population is rapidly increasing, with the number of older adults expected to reach 1.5 billion by 2050 (1). This demographic shift presents significant challenges in managing the health and well-being of elderly individuals, particularly in relation to chronic diseases that commonly arise with aging. Chronic conditions, including cardiovascular diseases, diabetes, and cognitive decline, are prevalent among older adults and are often exacerbated by poor oral health (2). Oral health is a crucial determinant of overall health in older populations, as it directly affects nutrition, social functioning, and systemic health (3).

The importance of oral health among older adults has received increasing attention in geriatric research. Oral diseases, such as periodontitis, dental caries, and gingival inflammation, are prevalent in aging individuals and can lead to significant functional impairments (4). Tooth loss, in particular, is one of the most common consequences of poor oral health in the elderly. Research has demonstrated that tooth loss is not only a marker of oral health but also a predictor of broader health outcomes, including malnutrition, frailty, and cognitive impairment (5, 6). Furthermore, tooth loss is linked to a decline in quality of life, as it affects speech, mastication, and overall self-esteem (7).

Recent research has increasingly emphasized the bidirectional relationship between oral health and systemic health in older adults (8). Chronic oral conditions, such as untreated periodontitis, have been associated with systemic inflammation, which may exacerbate the development of chronic diseases, including diabetes, cardiovascular disease, and even dementia. Conversely, the presence of these systemic diseases can further accelerate the deterioration of oral health, creating a vicious cycle that exacerbates both oral and general health. Therefore, maintaining oral health in older adults is essential not only for preserving dental function but also for preventing or mitigating the onset of other age-related diseases.

Tooth loss has been recognized as a significant risk factor for mortality in older populations (9). Several studies have documented that individual with fewer remaining teeth, or those with complete edentulism, face a higher risk of mortality compared to those with more teeth (10–12). A dose-response relationship has been observed, indicating that the more teeth a person loses, the greater their risk of death (13). Furthermore, recent studies have shown that rapid tooth loss, as opposed to gradual loss, is associated with greater functional disability (14). This finding points to the importance of understanding the trajectory of tooth loss over time, as it may provide valuable insights into the mechanisms through which oral health affects overall mortality risk.

Despite these findings, most studies have primarily focused on the relationship between tooth loss and mortality, typically examining only the baseline number of remaining teeth and its association with mortality outcomes. Few studies have investigated how tooth loss evolves over time or how these trajectories may impact all-cause mortality. The lack of longitudinal research in this area creates a gap in understanding the dynamic nature of oral health in aging populations. Therefore, the aim of this study is to investigate the trajectories of tooth loss over time and explore their association with all-cause mortality, addressing the critical question of whether distinct trajectories of tooth loss exist and how they affect mortality risk.

## 2 Method

### 2.1 Study population

Data for this study came from the Chinese Longitudinal Healthy Longevity Study (CLHLS), a longitudinal survey of older adults aged 65 years and above, as well as their adult children aged 35–64 years. Participants were categorized into two group based on survival status: surviving respondents and deceased individuals. The CLHLS collects data on a range of factors, including demographic characteristics, socio-economic background, family structure, health status, quality of life self-assessment, cognitive function, and personality and psychological characteristics. The survey also gathers information on the time and cause of death for deceased respondents. CLHLS employs a multistage cluster random sampling method, covering 866 counties and cities across 23 provinces in China. The survey was initially conducted in 1998, with follow-up surgeys in 2000, 2002, 2005, 2008–2009, 2011–2012, 2014, and 2017–2018. For further information, please visit the CLHLS website (https://opendata.pku.edu.cn/dataverse/CHADS). Written informed consent was obtained from all participants prior to their involvement in the study. The study was approved by the Peking University Biomedical Ethics Committee (IRB00001052-13074).

**Figure 1:**
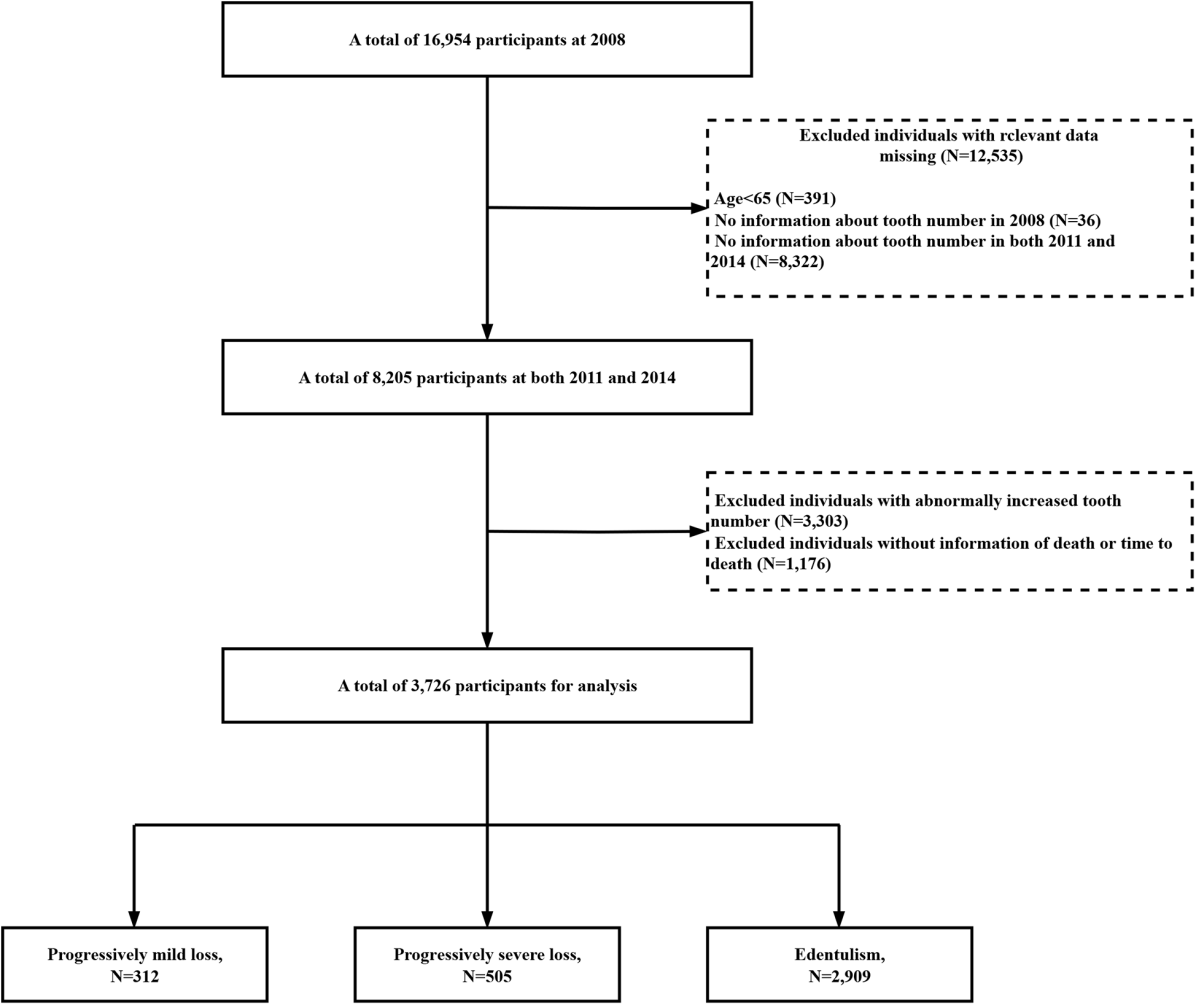
Flowchart of the sample selection process.

This study utilized longitudinal data from four waves of the CLHLS, beginning with the initial survey conducted in 2008–2009, followed by subsequent follow-ups in 2011–2012, 2014, and 2017–2018. Participants were excluded if they were younger than 65 years or had missing age data (*n* = 391), missing baseline data on tooth number (*n* = 36), missing tooth data at both the first and second follow-ups (*n* = 8,322), abnormally increased tooth number (*n* = 3,303), or missing death or time-to-death information (*n* = 1,176). Of the original cohort of 16,954 participants, ranging in age from 39 to 116 years, a total of 3,726 participants were included in the main analysis (Figure 1). To assess the potential impact of selection bias, we compared baseline characteristics and outcome prevalence between the analyzed and excluded participants, as shown in Supplementary Table 3. Additionally, the baseline characteristics of participants lost to follow-up and those included in the study are presented in Supplementary Table 4.

### 2.2 Assessment of teeth number

The number of natural teeth was assessed using the question, “How many natural teeth do you have? (excluding false teeth).” Participants who reported having 0 natural teeth were classified as having edentulism. To distinguish between edentulism and edentulism trajectory groups, all participants with edentulism were uniformly categorized into the “edentulism group.” Oral health data, including the number of natural teeth, were self-reported at baseline and during each follow-up every three years. Previous studies have confirmed that self-reported tooth number is a valid and reliable alternative to clinical examinations, as corroborated by comparative studies between clinical and self-reported data (15). Oral health status was self-reported by older adults, with interviewers available to assist participants in confirming the number of natural teeth and dentures used. Despite this, some individuals reported an abnormal increase in the number of natural teeth at subsequent surveys, which led to their exclusion from the analysis. An “abnormally increased tooth number” was defined as a reported increase in natural teeth exceeded the number reported in the previous survey wave. The three follow-up surveys, conducted in 2011–2012, 2014, and 2017–2018, utilized the same questionnaire and measurement methods as the baseline survey, ensuring consistency in data collection across all waves. For participants with exposure data in the first and second waves but missing data in the third wave, exposure was determined by establishing a trajectory using the first and second waves. Similarly, for participants with exposure data in the first and third waves, exposure was established using data from the first, second, and third waves (this methodology also applies to participants with data from only the first and third waves).

### 2.3 Assessment of all-cause mortality

Death was primarily determined based on death certificates provided by local authorities. In cases where a death certificate was unavailable, certificates supplied by the deceased's relatives were used. The definitions for outcome events, missing outcomes, and censoring were defined as follows: (1) Occurrence of outcome events: refers to individuals who clearly experienced an outcome event, such as death, during the study period; (2) Missing outcome events: refers to individuals who had missing death data in all subsequent follow-ups after trajectory grouping was identified, meaning they did not provide complete outcome event information during the study; (3) Censoring: refers to individuals who remained free of an outcome event during the follow-up closest to the study cutoff, meaning their outcome event status is unknown but no event occurred during the effective follow-up period after trajectory grouping was determined. In terms of outcome measurement timeline: (1) For participants with exposure data from the first and second waves but no data in the third wave, the second wave visit was used as the start of follow-up; (2) For participants with exposure data from the first and third waves, the third wave visit was used as the start of follow-up. For calculating follow-up time: (a) For participants with an outcome event, follow-up time was defined as the time at which the event occurred during follow-up minus the start of follow-up; (b) For censoring, follow-up time was defined as the time at the follow-up closest to the study cutoff minus the start of follow-up.

### 2.4 Assessment of covariates

Covariates were selected based on a thorough review of the literature, considering of factors that could potentially confound the relationship between tooth number and all-cause mortality. The selected covariates included age (continuous variable) (16), gender (16), education level (17), marital status (18), residence (16), household income (19), living state (20), Current exercise habits (21), smoking (22), drinking (23), BMI category (23), comorbidity (24), and cognitive impairment (25). Detailed information on these covariates is provided in Supplementary Table 1, while missing covariates are depicted in Supplementary Figure 1.

### 2.5 Statistical analysis

To address missing data in the CLHLS database, we applied the Template method (using the R package “VIM”) (26) and multiple imputation via Predictive Mean Matching (using the R package “mice”) (27). The imputation process consisted of five cycles (m = 5). A density plot was generated to compare the distributions of imputed data across both methods. The final imputed datasets were subsequently used for all analyses. Descriptive statistics are presented as mean (SD) for continuous variables and number (percentage) for categorical variables. We used the Kruskal–Wallis rank sum test for continuous variables and chi-square tests for categorical variables to compare baseline characteristics between the tooth trajectory groups identified through Growth Mixture Models (GMM). GMM was employed to explore different tooth number trajectories (28). GMM is a multilevel statistical technique that enables researchers to identify different distinct subpopulations within a population and estimate different growth trajectories for each subpopulation. GMM integrates the flexibility of multilevel modeling, which account for variation between individuals and time points, with latent class analysis, which identifies distinct subgroup within the population. This combination allows GMM to not only capture average growth trends at the group level but also to uncover and explain heterogeneity within the group.

The GMM was developed by initially fitting a single category and subsequently increasing the number of categories. Optimal models were selected based on the comparison of fit indices, interpretability, and practical significance. The Akaike Information Criteria (AIC), Bayesian Information Criteria (BIC), Sample Size Adjusted BIC (aBIC), entropy index, LoMendel-Rubin Likelihod Ratio Test (LMR), and Bootstrapped Likelihood Ratio Test (BLRT) were include in our study (29). Smaller value of AIC, BIC, and aBIC indicate better model fit. The LMR and BLRT are used to assess whether a significant difference exists between k and k-1 class models, with smaller the *p*-values suggesting that the k-class model is superior to the k-1 class model (30). Higher entropy value, closer to 1, reflect greater classification accuracy, meaning the model more accurately assigns individuals to the appropriate subpopulations (31). The fit indices presented in Supplementary Table 2 show a gradual decrease in information indices as the number of categories increases, making it difficult to determine the optimal model based solely on these indices. To address this, we utilized the scree plot for model selection (32), with the BIC results displayed in Supplementary Figure 2. The plot reveals three distinct inflection points, supporting the choice of a three-category model. After considering both statistical performance and clinical significance, we ultimately selected the three-category model for its interpretability and clinical significance in clinical settings.

Cox proportional hazards regression was used to assess the association between tooth number trajectories and the risk of all-cause mortality, reporting hazard ratios (HRs) with 95% confidence intervals (CIs). Three models were developed: Model 1 adjusted for age and gender; Model 2 was further adjusted for education level and marital status, while Model 3 included additionally adjustments for smoking, drinking, current exercise behavior, BMI category, comorbidities, cognitive impairment, and baseline number of teeth. To test the robustness of our findings, we performed a sensitivity analyses by excluding 453 participants with heart disease or stroke at baseline to mitigate potential bias from systemic diseases.

We also stratified all-cause mortality by several factors, including age (<80, ≥80), gender (male, female), residence (urban, rural), household income in the previous year (≤10,000, 10,000–≤50,000, >50,000), marital status (married or partnered, other marital status), education level (years) [no school (0 years), primary school (1–6 years), high school or above (at least 7 years)], living situation (with family members or in an institution, alone), smoking status (never, ever, current), drinking status (never, ever, current), current exercise (no, yes), BMI category (<18.5, 18.5–<24, ≥24), comorbidity (none, 1, ≥2), and cognitive impairment (no, yes). This stratification was performed to assess whether potential confounding variables influenced the association between Tooth Loss Trajectories and all-cause mortality and to examine interactions between tooth Loss trajectories and the stratified variables. Statistical analyses were conducted using R version 4.4.1 and EmpowerRCH version 4.0 software, with significance set at *p* < 0.05.

## 3 Result

### 3.1 Demographic and baseline characteristics

**Figure 2:**
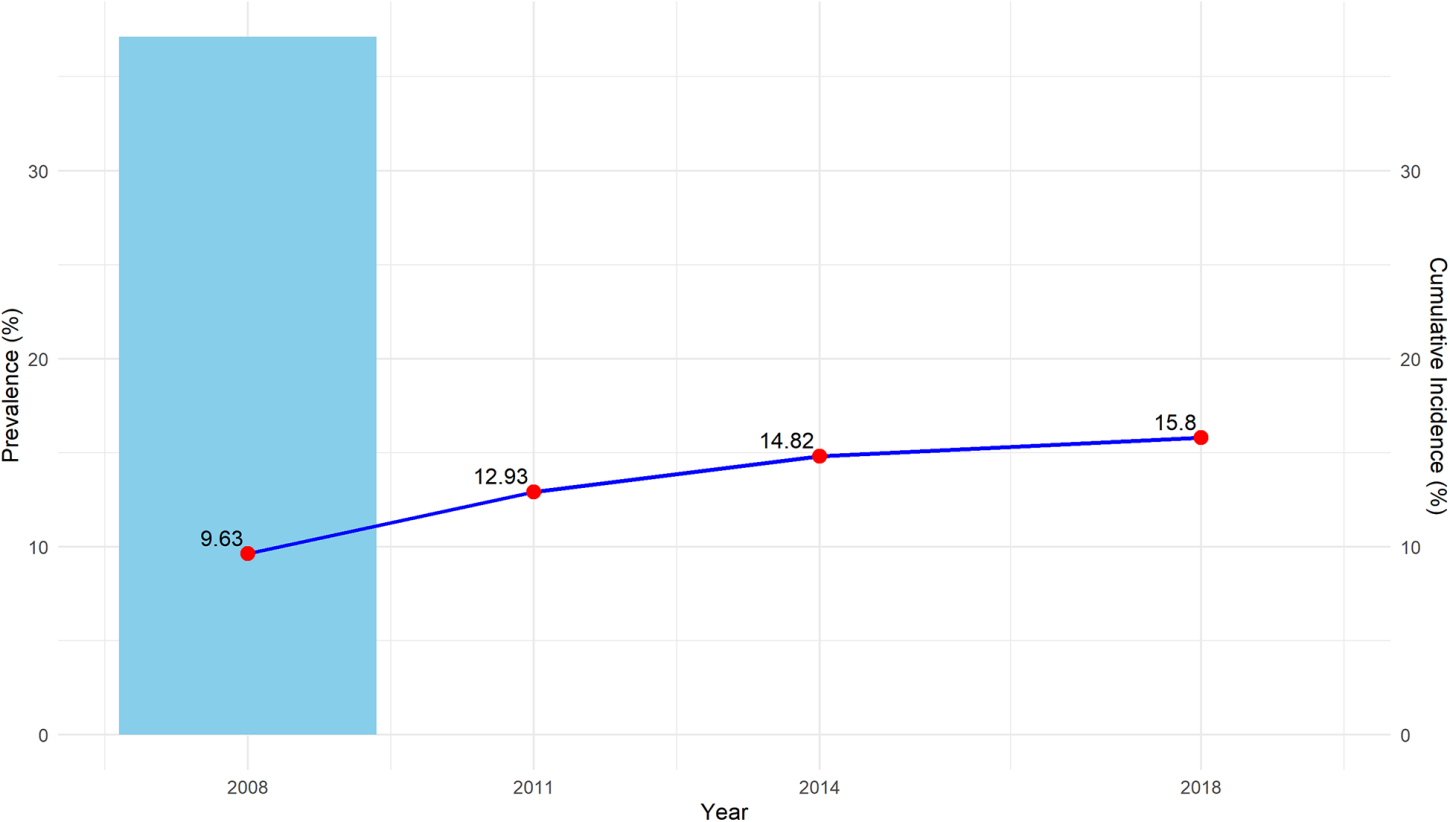
Prevalence and cumulative incidence of edentulism over time.

Table 1 summarizes the demographic and baseline characteristics of the participants, categorized by tooth number trajectories. A total of 3,726 participants were included in the final analysis, with 2,129 (57.14%) being female. The mean age was 85.16 ± 10.70 years, and the average BMI was 20.43 ± 3.51. A subset of 1,130 participants (30.33%) reported engaging in current exercise, while 698 (18.73%) were current drinkers and 701 (18.81%) were smokers. In terms of health conditions, 1,952 (52.39%) had no chronic diseases, 1,138 (30.54%) had one chronic diseases, and 636 (17.07%) had two or above of chronic diseases. Cognitive impairment was reported in 1,465 (39.32%) participants, and 2,672 (71.71%) had died from any cause by the end of the study. The prevalence of edentulism at the baseline survey in 2008 was 37.13%, and the cumulative incidence of edentulism over 10 years was 15.8%. In Figure 2, the blue box represents the baseline prevalence of edentulism in 2008, while the dark blue dashed lines indicate the cumulative incidence of edentulism over the 10-year period.

**Table 1:** Baseline characteristics of study participants by tooth loss trajectories in older adults in China, CLHLS 2008–2014 (*N* = 3,726).

Characteristics	Total (%)	Progressively mild loss (%)	Progressively severe loss (%)	Edentulism (%)	*P*-value
*N* (%)	3,726	312	505	2,909	
Age, years	85.16 ± 10.70	73.44 ± 7.79	78.01 ± 9.30	87.65 ± 9.76	<0.001
Gender					<0.001
Male	1,597 (42.86)	194 (62.18)	266 (52.67)	1,137 (39.09)	
Female	2,129 (57.14)	118 (37.82)	239 (47.33)	1,772 (60.91)	
Residence					0.016
Urban	2,454 (65.86)	183 (58.65)	330 (65.35)	1,941 (66.72)	
Rural	1,272 (34.14)	129 (41.35)	175 (34.65)	968 (33.28)	
Household income in the previous year (RMB)					0.004
≤10,000	2,131 (57.19)	155 (49.68)	283 (56.04)	1,693 (58.20)	
10,000–≤50,000	1,478 (39.67)	138 (44.23)	206 (40.79)	1,134 (38.98)	
>50,000	117 (3.14)	19 (6.09)	16 (3.17)	82 (2.82)	
Marital status, *n* (%)					<0.001
Married or partnered	2,433 (65.30)	87 (27.88)	245 (48.51)	2,101 (72.22)	
Other marital status	1,293 (34.70)	225 (72.12)	260 (51.49)	808 (27.78)	
Education level (years), *n* (%)					<0.001
No school	2,296 (61.62)	100 (32.05)	241 (47.72)	1,955 (67.21)	
Primary school	1,105 (29.66)	139 (44.55)	192 (38.02)	774 (26.61)	
High school and above	325 (8.72)	73 (23.40)	72 (14.26)	180 (6.19)	
Living state					<0.001
With family members or institution	3,147 (84.46)	291 (93.27)	423 (83.76)	2,433 (83.64)	
Alone	579 (15.54)	21 (6.73)	82 (16.24)	476 (16.36)	
Smoking					<0.001
No	2,473 (66.37)	163 (52.24)	317 (62.77)	1,993 (68.51)	
Ever	552 (14.81)	63 (20.19)	72 (14.26)	417 (14.33)	
Current	701 (18.81)	86 (27.56)	116 (22.97)	499 (17.15)	
Drinking					<0.001
No	2,563 (68.79)	189 (60.58)	315 (62.38)	2,059 (70.78)	
Ever	465 (12.48)	33 (10.58)	76 (15.05)	356 (12.24)	
Current	698 (18.73)	90 (28.85)	114 (22.57)	494 (16.98)	
Exercise at present					<0.001
No	2,596 (69.67)	174 (55.77)	317 (62.77)	2,105 (72.36)	
Yes	1,130 (30.33)	138 (44.23)	188 (37.23)	804 (27.64)	
BMI	20.43 ± 3.51	22.03 ± 3.89	21.25 ± 3.44	20.11 ± 3.41	<0.001
BMI category, *n* (%)					<0.001
<18.5	1,187 (31.86)	49 (15.71)	120 (23.76)	1,018 (34.99)	
18.5–<24	2,007 (53.86)	183 (58.65)	282 (55.84)	1,542 (53.01)	
≥24	532 (14.28)	80 (25.64)	103 (20.40)	349 (12.00)	
Comorbidity					<0.001
None	1,952 (52.39)	133 (42.63)	260 (51.49)	1,559 (53.59)	
1	1,138 (30.54)	96 (30.77)	158 (31.29)	884 (30.39)	
2 or above	636 (17.07)	83 (26.60)	87 (17.23)	466 (16.02)	
Cognitive impairment					<0.001
No	2,261 (60.68)	271 (86.86)	398 (78.81)	1,592 (54.73)	
Yes	1,465 (39.32)	41 (13.14)	107 (21.19)	1,317 (45.27)	
Number of teeth	8.51 ± 9.98	27.78 ± 3.44	19.83 ± 6.18	4.48 ± 6.43	<0.001
Death					<0.001
No	1,054 (28.29)	198 (63.46)	231 (45.74)	625 (21.49)	
Yes	2,672 (71.71)	114 (36.54)	274 (54.26)	2,284 (78.51)	

Continuous variables were expressed as mean ± standard deviation (SD) in case of normal distribution and compared between two groups by Kruskal–Wallis rank sum test. If the count variable had a theoretical number <10, Fisher's exact probability test was used. Categorical variables are presented as counts (percentages) and compared by Chi-square test.

Compared to participants with progressively mild tooth loss, those in the progressively severe loss and edentulism groups were older, had a lower BMI, were more likely to be female, resided in urban areas, had lower incomes, were married, and had lower rates of cohabitation with family members or institutionalized care. This group also exhibited lower rates of smoking, drinking, and exercisers, but had a higher prevalence of hypertension and cognitive impairment. The characteristics and outcome prevalence between the analyzed and excluded participants are shown in Supplementary Tables 3 and 4. No significant differences were found in gender, living status, BMI category, and comorbidity between the analyzed and excluded participants, However, differences were found in other characteristics and outcome prevalence (*p* < 0.05). Except for age, residence, income, marital status, and cognitive impairment, the standardized mean difference (SMD) for other characteristics was less than 0.1. For example, the SMD for death was 0.04 (range 0.00–0.08), with 73.56% of excluded participants and 71.71% of those in the study having died, representing 7,076 and 2,672 participants, respectively.

### 3.2 Trajectory models for tooth lost

**Figure 3:**
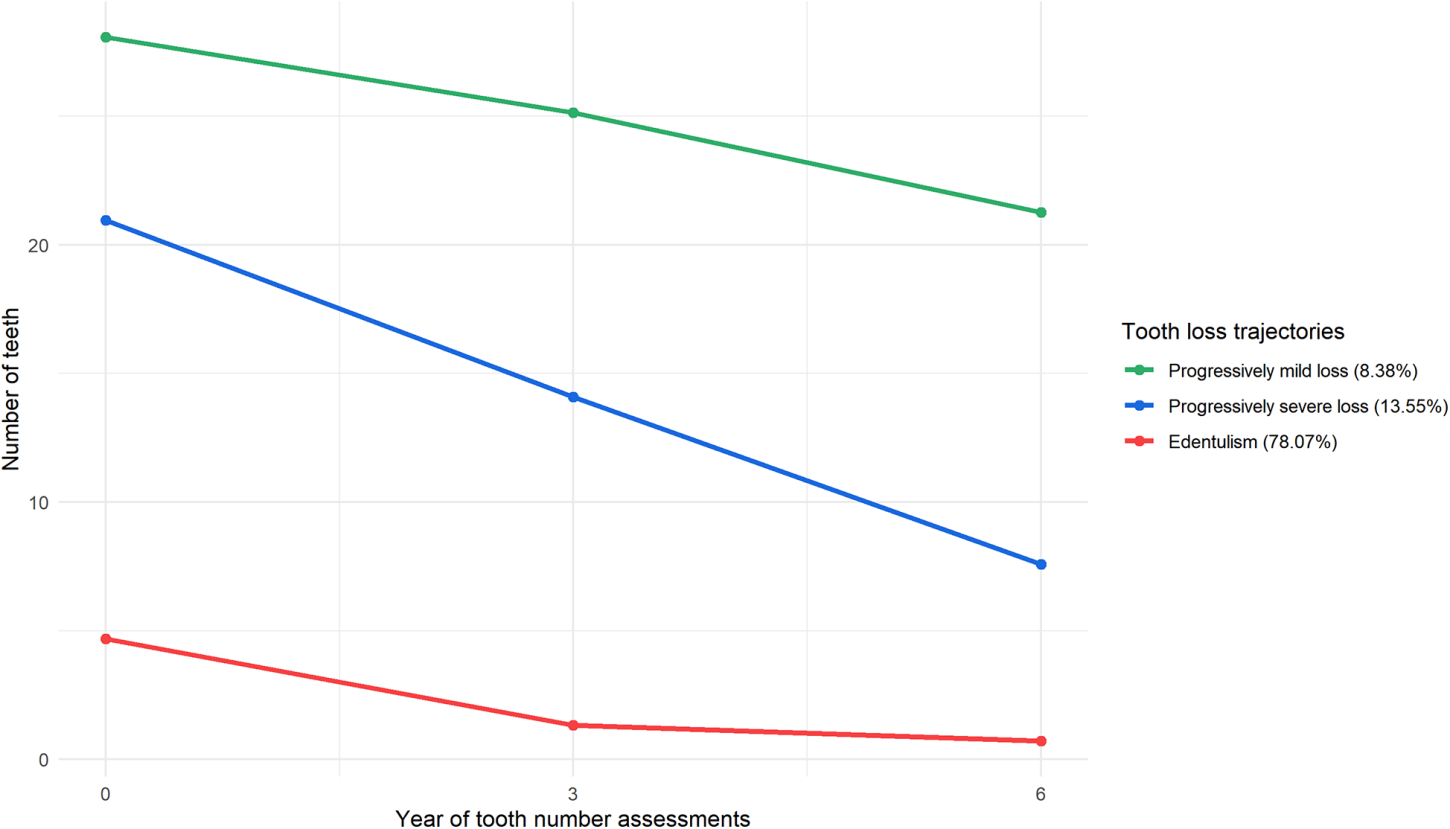
Tooth loss trajectories among older Chinese adults, CLHLS 2008–2014 (*N* = 3,726).

The Growth Mixture Model (GMM) analysis identified distinct latent classes based on the trajectory of tooth loss, with varying classifications. As shown in Supplementary Table 2, the likelihood ratio tests (LMR and BLRT) indicated that the best models were those with 3, 4, and 5 classes. However, because BIC decreases as the number of classes increases without reaching a minimum value, we plotted lithotripsy curve to determine the optimal number of classes (Supplementary Figure 2). The 3-class model was selected as the final model due to its superior fit [AIC = 55,159.909, BIC = 55,247.032, ABIC = 55,202.547, Entropy = 0.959, LMR (p) < 0.001, BLRT (p) < 0.001]. Over 9.41 years of follow-up, three distinct trajectory groups were identified: (1) progressively mild loss (312 participants, 8.37%), (2) progressively severe loss (505 participants, 13.55%), and (3) edentulism (2,909 participants, 78.07%) as shown in Figure 3. The median follow-up times for the progressively mild loss, progressively severe loss, and edentulism groups were 5.91, 3.44, and 1.84 years, respectively. The survival curves across the three groups reveal significant differences in outcome prevalence (*P* < 0.0001 for all comparisons), as shown in Supplementary Figure 4. The edentulism group exhibited the highest prevalence of outcomes, while the progressively mild loss group showed the lowest. The number of deaths in each group was as follows: progressively mild loss, 114 (36.54%); progressively severe loss, 274 (54.26%); and edentulism, 2,284 (78.51%). The mean age (±SD) for the participants in the progressively mild loss, progressively severe loss, and edentulism groups was 73.44 ± 7.79, 78.01 ± 9.30, and 87.65 ± 9.76 years, respectively, as shown in Table 1. Among those who died, the mean age (±SD) at death for each trajectory group was 77.48 ± 9.08, 81.31 ± 9.32, and 90.8 ± 8.56 years, respectively.

### 3.3 Univariate and multivariate analysis of all-cause mortality

Table 2 presents the results of the univariate analysis conducted over the the period from 2008 to 2018. Significant associations were observed between all-cause mortality and several variables, including age, marital status, education level, smoking, drinking, exercise, Body Mass Index (BMI) category, comorbidity, and cognitive impairment. Although gender did not show a significant univariate association with mortality, it was included as a confounder in the multivariate models. Univariate analysis adjusted for age is presented in Supplementary Table 5.

**Table 2:** Univariate analysis Cox regression analysis associated with all-cause mortality.

*N* (%)	All-cause mortality		
	Statistics	HR (95% CI)	*P*-value
Age, years	85.16 ± 10.70	1.06 (1.06, 1.07)	<0.0001
Gender			
Male	1,597 (42.86%)	1.0	
Female	2,129 (57.14%)	1.03 (0.96, 1.12)	0.3864
Residence			
Urban	2,454 (65.86%)	1.0	
Rural	1,272 (34.14%)	1.02 (0.94, 1.10)	0.6434
Household income in the previous year (RMB)			
≤10,000	2,131 (57.19%)	1.0	
10,000–≤50,000	1,478 (39.67%)	1.04 (0.96, 1.12)	0.3760
>50,000	117 (3.14%)	0.98 (0.78, 1.22)	0.8252
Marital status, *n* (%)			
Married or partnered	2,433 (65.30%)	1.0	
Other marital status	1,293 (34.70%)	0.53 (0.49, 0.58)	<0.0001
Education level (years), *n* (%)			
No school (0 years)	2,296 (61.62%)	1.0	
Primary school (1–6 years)	1,105 (29.66%)	0.75 (0.68, 0.81)	<0.0001
High school and above (at least 7 years)	325 (8.72%)	0.64 (0.55, 0.75)	<0.0001
Living state			
With family members or institution	3,147 (84.46%)	1.0	
Alone	579 (15.54%)	0.93 (0.83, 1.03)	0.1589
Smoking			
No	2,473 (66.37%)	1.0	
Ever	552 (14.81%)	1.10 (0.99, 1.22)	0.0909
Current	701 (18.81%)	0.82 (0.74, 0.91)	0.0002
Drinking			
No	2,563 (68.79%)	1.0	
Ever	465 (12.48%)	1.03 (0.92, 1.15)	0.6372
Current	698 (18.73%)	0.88 (0.80, 0.97)	0.0138
Exercise at present			
No	2,596 (69.67%)	1.0	
Yes	1,130 (30.33%)	0.81 (0.74, 0.88)	<0.0001
BMI category, *n* (%)			
<18.5	1,187 (31.86%)	1.0	
18.5–<24	2,007 (53.86%)	0.78 (0.72, 0.85)	<0.0001
≥24	532 (14.28%)	0.59 (0.52, 0.68)	<0.0001
Comorbidity			
None	1,952 (52.39%)	1.0	
1	1,138 (30.54%)	0.97 (0.89, 1.06)	0.5345
2 or above	636 (17.07%)	0.84 (0.76, 0.94)	0.0020
Cognitive impairment			
No	2,261 (60.68%)	1.0	
Yes	1,465 (39.32%)	1.97 (1.82, 2.13)	<0.0001
Number of teeth	8.51 ± 9.98	0.97 (0.96, 0.97)	<0.0001

Continuous variables were expressed as mean ± standard deviation (SD); categorical variables are presented as counts (percentages).

After adjusting for age (continuous) and gender, cox regression analysis indicated that both the progressively severe loss and edentulism groups had significantly higher risks of all-cause mortality compared to the progressively mild loss group (HR for progressively severe loss = 1.27, 95% CI: 1.00–1.60; HR for edentulism = 1.63, 95% CI: 1.30–2.01). After adjusting for potential confounders, including age (continuous), gender, education level, marital status, smoking, drinking, current exercise, BMI category, comorbidity, cognitive impairment, and baseline number of teeth, the hazard ratios (HRs) for the progressively severe loss and edentulism groups increased further (HR = 1.29, 95% CI: 1.01–1.64; HR = 1.60, 95% CI: 1.28–1.99, respectively), as shown in Table 3. Consistent results were also observed in the sensitivity analysis (Supplementary Table 6).

**Table 3:** Multiple Cox regression analysis association (HRs, 95% CI) between tooth loss trajectories and all-cause mortality.

Variables	Model I		Model II		Model III	
	HR (95% CI)	*P*-value	HR (95% CI)	*P*-value	HR (95% CI)	*P*-value
Tooth loss trajectories						
Progressively mild loss	Ref		Ref		Ref	
Progressively severe loss	1.27 (1.00, 1.60)	0.046	1.24 (0.98, 1.57)	0.069	1.29 (1.01, 1.64)	0.039
Edentulism	1.63 (1.32, 2.01)	<0.001	1.59 (1.29, 1.96)	<0.001	1.60 (1.28, 1.99)	<0.001
*P* for trend	<0.0001		<0.0001		<0.0001	
Age, years					1.06 (1.05, 1.06)	<0.001
Gender						
Male					Ref	
Female					0.75 (0.67, 0.83)	<0.001
Marital status, *n* (%)						
Married or partnered					Ref	
Other marital status					0.94 (0.85, 1.05)	0.270
Education level, *n* (%)						
No school					Ref	
Primary school					1.01 (0.91, 1.12)	0.871
High school and above					1.03 (0.87, 1.21)	0.746
Smoking						
No					Ref	
Ever					1.18 (1.05, 1.34)	0.007
Current					1.01 (0.89, 1.13)	0.919
Drinking						
No					Ref	
Ever					1.00 (0.88, 1.13)	0.995
Current					0.98 (0.88, 1.10)	0.774
Exercise at present						
No					Ref	
Yes					0.95 (0.87, 1.04)	0.293
BMI category, *n* (%)						
<18.5					Ref	
18.5 to <24					1.02 (0.94, 1.11)	0.599
≥24					0.92 (0.80, 1.05)	0.205
Comorbidity						
None					Ref	
1					1.08 (0.99, 1.17)	0.100
2 or above					1.06 (0.95, 1.18)	0.310
Cognitive impairment						
No					Ref	
Yes					1.18 (1.09, 1.29)	<0.001
Baseline number of teeth					1.00 (0.99, 1.01)	0.9443

BMI: Body Mass Index.

Model I, adjust for age (continuous) and gender.

Model II, adjust for age (continuous), gender, education level and marital status.

Model III, adjust for age (continuous), gender, education level, marital status smoking, drinking, exercise at present, BMI category, comorbidity, cognitive impairment, and baseline number of teeth.

Results of Multiple cox regression analysis were presented as Hazard Ratio (HRs) and 95% confidence intervals (CIs).

Subgroup analyses based on the three tooth loss trajectory groups revealed no interaction between tooth loss trajectories and the subgroup variables (Supplementary Figure 3).

## 4 Discussion

This study is the first to examine the association between tooth loss trajectories and all-cause mortality in older Chinese adults. Three distinct trajectories were identified: Progressively Mild Loss (8.24%), Progressively Severe Loss (14.08%), and Edentulism group (77.69%). Compared to individuals with progressively mild tooth loss, those with progressively severe loss or edentulism group were found to have a significantly higher risk of all-cause mortality. These findings emphasize the importance of maintaining oral health and preserving natural teeth to promote longevity among older adults.

At baseline, the prevalence of edentulism in this study was 37.13%. In contrast, previous studies utilizing the China Health and Retirement Longitudinal Study (CHARLS) database reported a prevalence of 15.4% among adults aged 45 and older and 30.5% among the elderly population (33). The discrepancy is likely due to the higher proportion of very elderly individuals in the CLHLS cohort compared to CHARLS. Generally, the prevalence of edentulism increases with age. The cumulative incidence of edentulism over 10 years was 15.8%, a figure of notable public health significance. While high-income countries,such as the United States, have seen a decline in edentulism due to better access to dental care and public health initiatives (34), the prevalence remain high in low- and middle-income countries, including China, particularly in rural and underserved areas. Disparities in dental care access, oral health education, and preventive measures likely contribute to the elevated rates observed in this study. Addressing these disparities requires targeted public health initiatives to enhance oral health awareness and improve access to affordable dental care services, particularly for older adults.

The novel contribution of this study lies in its dynamic depiction of tooth loss trajectories, which we categorized into three distinct patterns: slow decline, rapid decline, and edentulism. While previous research has predominantly focused on baseline tooth count and its correlation with mortality (9, 10), our trajectory-based approach reveals that individuals experiencing rapid tooth loss or edentulism are at a significantly higher risks of all-cause mortality, with the edentulism group exhibiting the highest risk. These findings emphasize the critical importance of early interventions aimed at stabilizing tooth count, which plays a critical role in promoting healthy aging. According to the WHO, retaining at least 20 teeth significantly reduces the risk of adverse clinical outcomes in older populations. Our results are consistent with recent studies (14) linking tooth loss trajectories to negative health outcomes, further highlighting the significance of oral health in supporting overall well-being among older adults.

When comparing our study to Huang et al.'s work, we noted several differences and similarities in the trajectories. Huang et al. Classified participants into four trajectories, while we identified three. This discrepancy may stem from their failure to account for participants with abnormally increased tooth numbers, which likely lead to a larger sample and a different categorization approach. Additionally, Huang et al.'s study may did not clearly delineate the exposure and outcome measurement periods, which resulted in an overlap between the long-term exposure and outcome data. This methodological limitation may complicates the interpretation of reverse causality. Despite these methodological differences, both studies found significant heterogeneity in tooth loss trajectories among older populations.

Tooth loss and edentulism significantly increase the risk of mortality through several interconnected mechanisms. A key factor is impaired masticatory function, which restricts edentulous individuals' ability to maintain a balanced diet, particularly with respect to consuming fiber- and vitamin-rich foods such as fruits, vegetables, and meat (35). These dietary deficiencies lead to inadequate intake of essential nutrients necessary for healthy aging and the prevention of chronic diseases (36). Furthermore, poor oral health in edentulous individuals disrupts the oral microbiota, increasing pathogenic bacteria while reducing beneficial microbes. This imbalance fosters chronic inflammation and bacteremia, thereby exacerbating systemic inflammation, a well-established risk factor for cardiovascular diseases (CVD), which are strongly associated with elevated all-cause mortality (37, 38). In addtion, prosthodontic treatments, including dentures and implants, may introduce potential risks. In China, many older adults rely on low-cost dental services of variable quality, which may result in complications such as infections and implant failures. Even in well-established medical facilities, the surgical risks for elderly patients remain substantial, further influencing long-term health outcomes and mortality (39). Moreover, the co-occurrence of tooth loss with aging-related multimorbidity amplifies the burden of chronic diseases and functional decline, which contributes to an elevated mortality risk. Tooth loss may also reflect the aging process itself, which is intrinsically linked to progressive health deterioration. Socioeconomic factors further exacerbate these risks. Lower socioeconomic status is associated with inadequate access to dental care, reduced health literacy, and disparities in healthcare services. Additionally, tooth loss may reduce social status and income, creating a feedback loop that intensifies health risks and mortality (40). Finally, edentulism has a negative impact on psychosocial well-being, including self-esteem and social engagement. Affected individuals often experience social withdrawal and depression, further diminishing their quality of life and increasing their risk of mortality. Consequently, tooth loss and its associated sequelae render older adults a high-risk population for all-cause mortality.

Our study provides several important clinical implications. First, we observed a significantly increased risk of all-cause mortality in the edentulism and progressively severe tooth loss groups compared to the progressively mild loss group, highlighting the significant association between tooth loss trajectories and all-cause mortality. While previous literature has demonstrated that tooth loss impairs masticatory function and negatively affects mental health and social participation (3), our findings underscore the urgent need for preventive measures. Public health initiatives should prioritize promoting good oral hygiene practices, such as regular brushing with fluoridated toothpaste, interdental cleaning, and routine dental check-ups. Moreover, maintaining overall oral and nutritional health may offer more benefits than focusing solely on tooth retention. Second, the prevalence of edentulism in our study was 37.13%, indicating a high cumulative incidence and a significant proportion of older adults at risk for developing edentulism. These findings highlight the importance of addressing edentulism as a public health concern. In addition to its impact on oral function, edentulism has been linked to increased risks of cardiovascular multimorbidity and all-cause mortality (23). Strengthened oral health education and implementing early intervention strategies by healthcare providers could help prevent the progression of edentulism. Active management of oral health may, therefore, serve as an effective strategy to mitigating mortality risks. Finally, our subgroup analysis showed no statistical interaction between tooth loss trajectories and the risk of all-cause mortality, suggesting that the observed results are generally applicable across different subgroups. Notably, older adults over 80 years of age, those with lower income, lower education levels, current drinking habits, overweight, two or more chronic diseases, or cognitive impairment face higher mortality risks. These elevated risks may be attributed to barriers such as mobility limitations, insufficient oral health knowledge, or cognitive decline. Socioeconomic disadvantages are also strongly associated with higher rates of dental caries (23), a key risk factor for edentulism. Therefore, governments and communities should implement targeted support, such as door-to-door oral care, specialized educational programs, and financial assistance, to help vulnerable populations improve their oral health. For individuals with cognitive impairment or mobility challenges, family support in arranging regular check-ups and treatment, as well as tailored oral care guidance, can significantly enhance healthy aging outcomes. While current evidence from long-term studies on tooth loss and all-cause mortality remains limited, future research involving diverse populations and longitudinal cohorts may yield more robust insights. Such studies could ultimately support the notion of maintaining natural teeth as a key determinant of a healthier and longer lifespan for older adults.

This study has several notable strengths. It identifies distinct tooth loss trajectories and their associations with all-cause mortality, providing valuable insights into disease progression and its long-term implications. Additionally, the study's large, nationally representative sample, with up to 10 years of follow-up, may strengthens its generalizability to the broader population of older Chinese adults. However, several limitations must be acknowledged. First, oral health conditions were self-reported, which may introduce inaccuracies. Although interviewers assisted participants in verifying the number of natural teeth and dentures, some individuals inaccurately reported increases in their number of natural teeth during follow-up, resulting in their exclusion from the analysis. This exclusion may have reduced the sample size and potentially affected the stability of the results. Fortunately, comparisons of baseline characteristics and outcome prevalence between the analyzed and excluded participants offer valuable evidence to assess the potential impact of selection bias. Second, the factors examined in our study are often interconnected with aging and systemic health, which could potentially contribute to mortality and introduce confounding effects. Third, our study specifically focused on natural teeth and did not consider dentures, which may also influence both oral health and overall mortality. Finally, as an observational study, residual confounding cannot be entirely ruled out, despite our efforts to adjust for potential biases in the analysis.

## 5 Conclusion

In conclusion, our study confirms three distinct tooth loss trajectories among Chinese older adults, with edentulism showing the strongest association with all-cause mortality. Our findings suggest that maintaining oral health and preventing tooth loss are essential for enhancing quality of life and promoting healthy aging. Edentulism, in particular, emerges as a significant public health concern, not only due to its impact on oral function but also its association with increased risks of cardiovascular disease (CVD), multimorbidity, and mortality. To address these challenges, public health initiatives should prioritize improving oral health education and ensuring access to dental care, especially for older populations. Community-based programs that offer regular dental check-ups, preventive care, and early interventions can help mitigate tooth loss. Special attention should be given to vulnerable populations, including those with low socioeconomic status, cognitive impairment, or limited access to healthcare. Public health campaigns should aim to raise awareness about the importance of oral hygiene and the role it plays in overall health, alongside providing targeted interventions to reduce the risk of tooth loss and its associated outcomes. While our study contributes valuable insights into the relationship between tooth loss and mortality, further research, especially long-term studies in diverse populations, is essential to establish definitive causal links and inform more effective public health strategies for promoting healthier aging.

## Data Availability

The original contributions presented in the study are included in the article/Supplementary Material, further inquiries can be directed to the corresponding author.
