# Supplementary material for: Tooth loss trajectories and their association with all-cause mortality among older Chinese adults
Source: Front Oral Health. 2025 Feb 26;6:1535708. doi: 10.3389/froh.2025.1535708 (PMC11897258; doi:10.3389/froh.2025.1535708)
Supplement: Supplementary file 1 [file Table1.docx]

**Supplementary Material:**

**Legend**

**Supplementary Figure 1.** Proportion of missing variables in the total population.

**Supplementary Figure 2.** The Scree Plot of GMM Presented BIC Values by class.

**Supplementary Figure 3.** The association between tooth Loss trajectories and all-cause mortality, stratified by participant characteristics within Cox regression based on Model 3.

**Supplementary Figure 4.** The Kaplan-Meier survival curves for all-cause mortality.

**Supplementary Table 1.** Descriptions of covariates.

**Supplementary Table 2.** Model fit information for teeth lost trajectories in 3726 participants.

**Supplementary Table 3.** The characteristics and outcome prevalence between the analyzed and excluded participants

**Supplementary Table 4.** Characteristics of participants lost to follow-up versus participants in the final study.

**Supplementary Table 5.** Univariate analysis Cox regression analysis associated with all-cause mortality.

**Supplementary Table 6.** Sensitivity analysis by excluding participants (N = 453) with heart diseases or stroke at baseline.


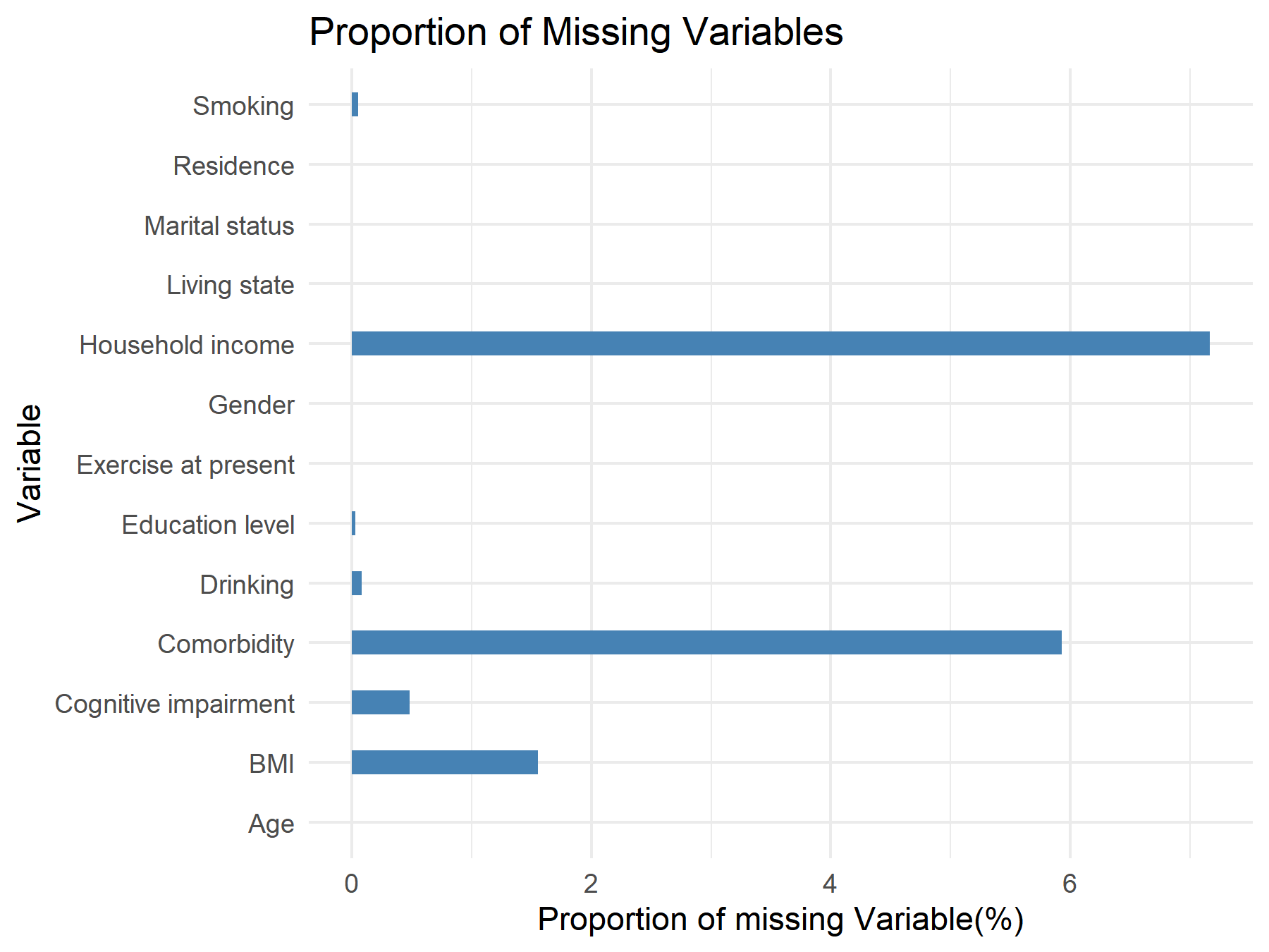


**Supplementary Figure 1.** Proportion of missing variables in the total population.


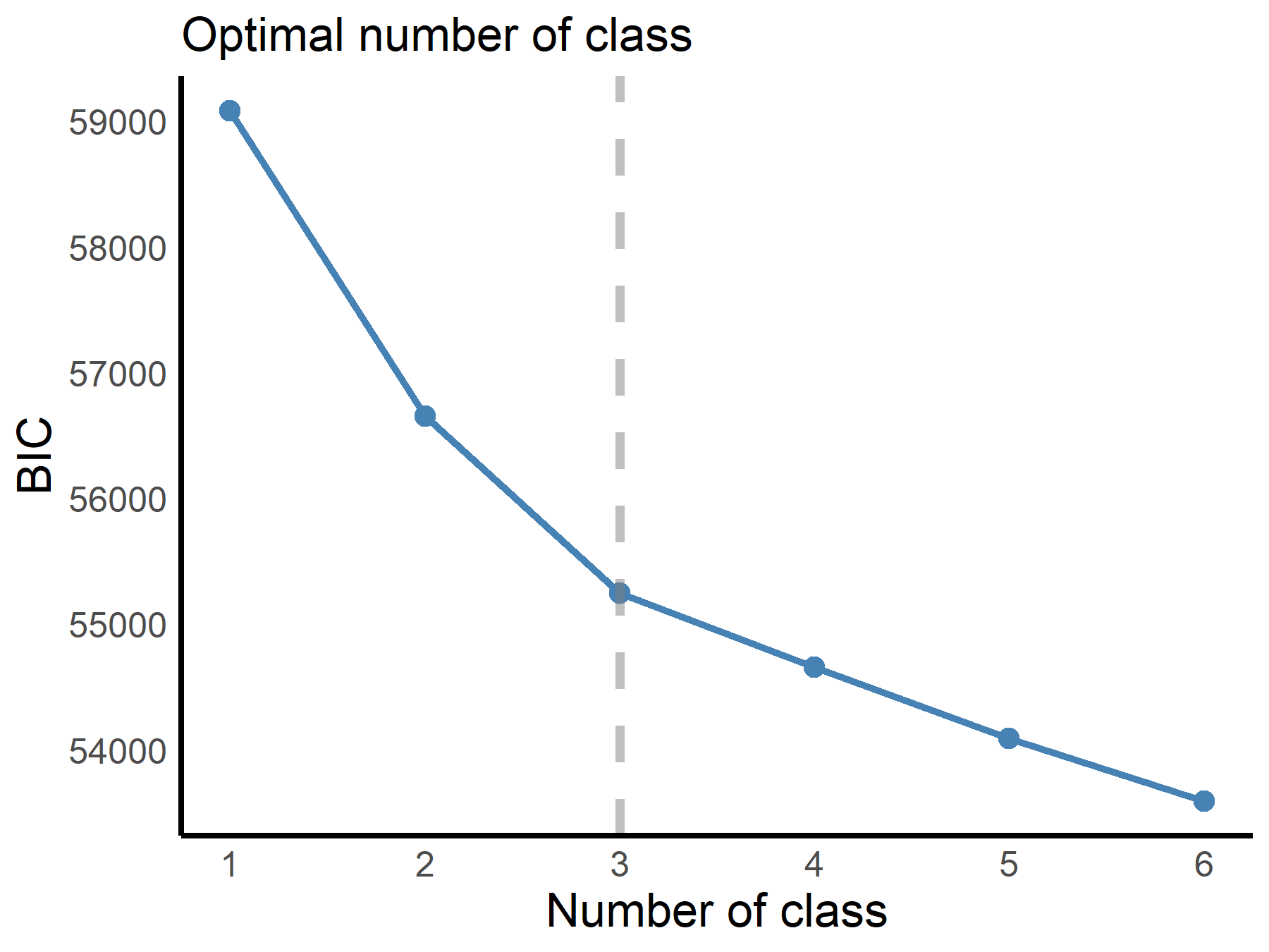


**Supplementary Figure 2.** The Scree Plot of GMM Presented BIC Values by class.


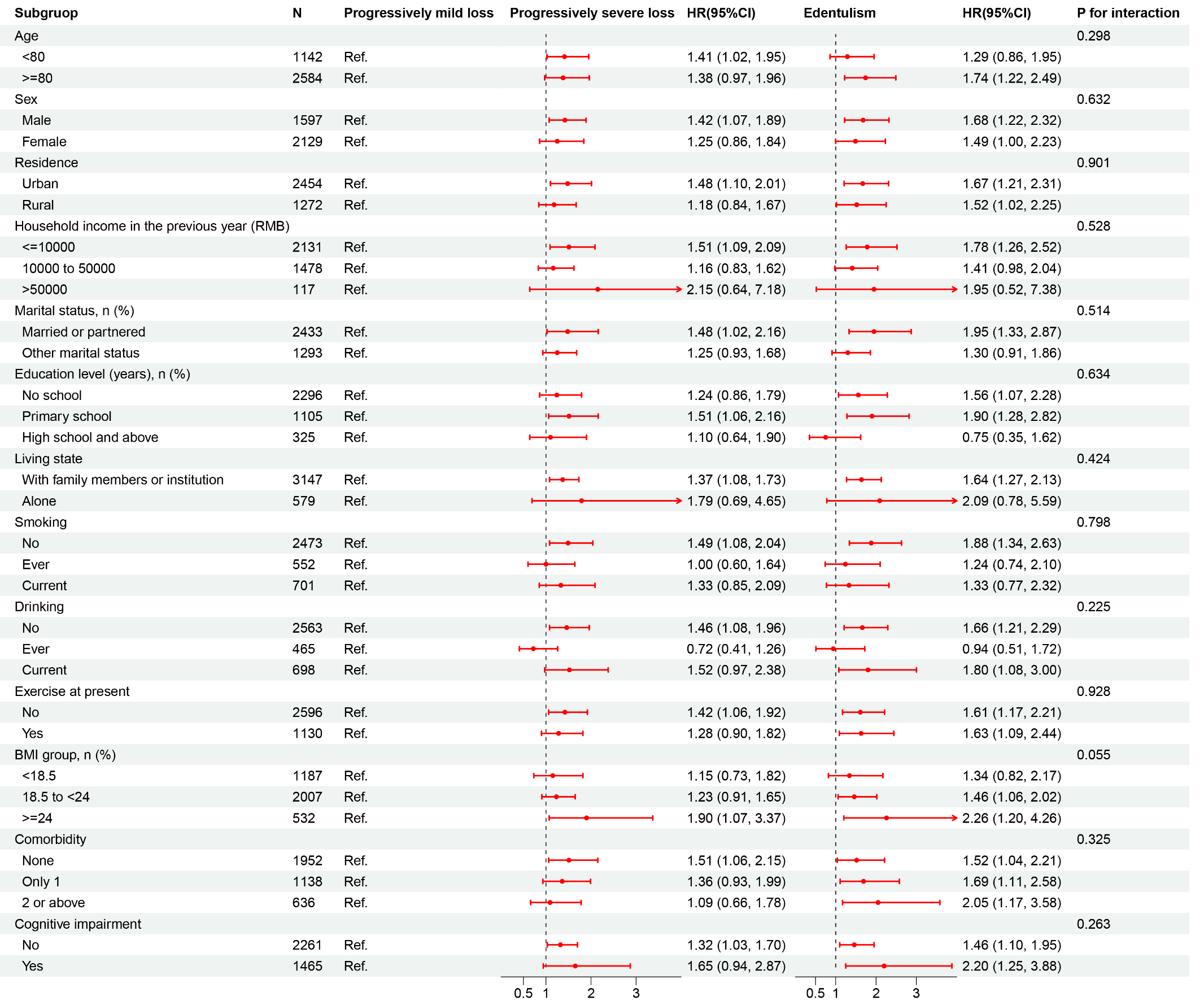


**Supplementary Figure 3.** The association between tooth Loss trajectories and all-cause mortality, stratified by participant characteristics within Cox regression based on Model 3.


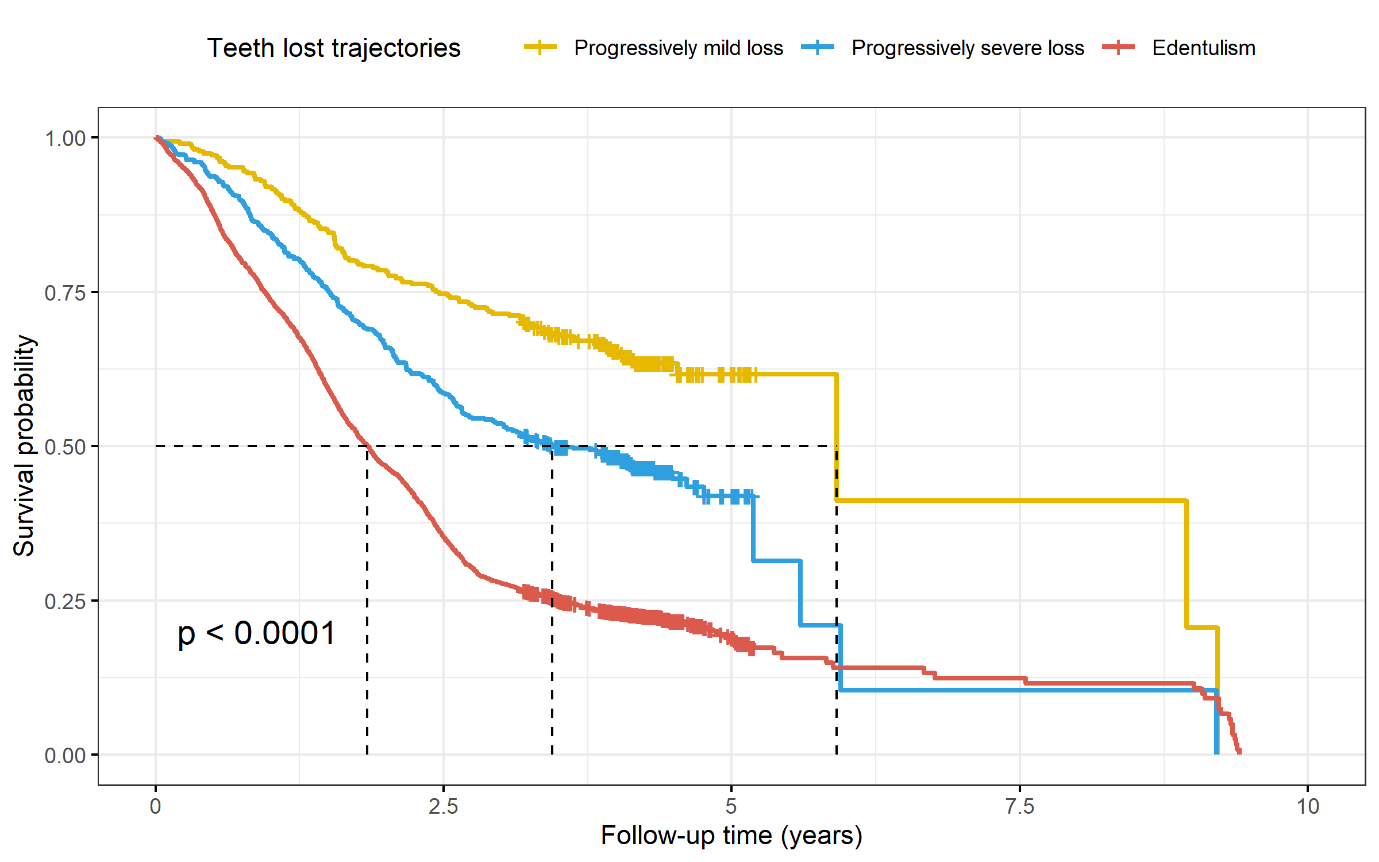


**Supplementary Figure 4.** The Kaplan-Meier survival curves for all-cause mortality.

| **Supplementary Table S1 Descriptions of covariates** | |
| --- | --- |
| **Variables** |  |
| **Age** | validated age, group by age < 80, ≥80 years |
| **Gender** | Gender represents biological sex, included male and female |
| **Residence** | category of residence of the interviewee in 2008 |
| **Household income** | total income of your household last yeare (< 10,000/10000 to 50,000/> 50,000 yuan) |
| **Marital status** | current marital status(married and living with a spouse/others) |
| **Education level** | Education level was categorized into three groups in this study to obtain robust results: no schooling (0 years), primary school (1–6 years), and high school or above (at least 7 years). |
| **Living state** | co-residence of interviewee (With family members or institution/Alone) |
| **Smoking** | Smoking was defined as never smoking, ever smoking, and current smoking behavior. |
| **Drinking** | Drinking was defined as never drinking, ever drinking, and current drinking behavior. |
| **Exercise at present** | Exercise at present is a binary variable divided into no and yes |
| **BMI** | Body mass index (BMI) was calculated as weight (kg) divided by the square of height (m²), with categories defined as: <18.5, 18.5 to <24, and ≥24 kg/m². |
| **Comorbidity** | 11 chronic diseases including hypertensive, diabetes, heart diseases, stroke, cancer, arthritis, dyslipidemia, hepatitis, chronic nephritis, gastric or duodenal ulcer, and asthma were based on self-reported disease histories collected from questionnaires. Number of chronic diseases was considered as a covariate (none/only 1 chronic disease/2 and more chronic diseases) |
| **Cognitive impairment** | The Mini-Mental State Examination (MMSE) is a scale that assesses participants' cognitive function and contains 24 items divided into 7 different domains: orientation (5 points), food naming (7 points), memorized words (3 points), attention and calculation (5 points), figure reproduction (1 point), memory recall (3 points), and language ability (6 points). MMSE scores range from 0 to 30, with higher scores indicating stronger cognitive functioning in individuals. In the assessment, if participants failed to give a response, we regarded this as a wrong answer. We considered a total score of 24 or less as having cognitive impairment according to the MMSE scoring criteria. |

| **Supplementary Table 2**. Model fit information for teeth lost trajectories in 3726 participants. | | | | | | | | |
| --- | --- | --- | --- | --- | --- | --- | --- | --- |
| classes | AIC | BIC | aBIC | LMR(p) | BLRT(p) | Entropy | Group size(N)^#^ | Group size(%)^#^ |
| 1 | 59033.253 | 59083.037 | 59057.617 | NA | NA | NA | 3726 | 100 |
| 2 | 56588.335 | 56656.789 | 56621.836 | < 0.001 | < 0.001 | 0.961 | 507 / 3219 | 13.61 / 86.39 |
| **3** | **55159.909** | **55247.032** | **55202.547** | **0.0017** | **< 0.001** | **0.959** | **312 / 505 / 2909** | **8.37 / 13.55 / 78.07** |
| 4 | 54551.075 | 54656.867 | 54602.850 | 0.0101 | < 0.001 | 0.925 | 224 / 319 / 2760 / 423 | 6.01 / 8.56 / 74.07 / 11.35 |
| 5 | 53964.030 | 54088.492 | 54024.942 | 0.1286 | < 0.001 | 0.942 | 259 / 90 / 309 / 2823 / 245 | 6.95 / 2.42 / 8.29 / 75.77 / 6.58 |
| 6 | 53448.878 | 53592.009 | 53518.926 | 0.0097 | < 0.001 | 0.947 | 106 / 220 / 67 / 208 / 2801 / 324 | 2.85 / 5.9 / 1.8 / 5.58 / 75.17 / 8.7 |

Bold fonts belong to the best class.

aBIC – adjusted Bayesian information criterion, AIC – Akaike’s information criterion, BIC – Bayesian information criterion, BLRT – bootstrapped likelihood ratio test, LMR – Vuong-Lo-Mendell–Rubin test, NA – not applicable.

^#^ Group size represents the sample size in each trajectory group based on number of latent classes.

| **Supplementary Table 3**. The characteristics and outcome prevalence between the analyzed and excluded participants | | | | | |
| --- | --- | --- | --- | --- | --- |
| Characteristics | Total (%) | Excluded (%) | Included (%) | Standardize diff. | P-value |
| N (%) | 16954 | 13228 | 3726 |  |  |
| Age, years | 86.85 ± 11.97 | 87.33 ± 12.26 | 85.16 ± 10.70 | 0.19 (0.15, 0.22) | <0.001 |
| Gender |  |  |  | 0.00 (-0.03, 0.04) | 0.904 |
| Male | 7252 (42.77) | 5655 (42.75) | 1597 (42.86) |  |  |
| Female | 9702 (57.23) | 7573 (57.25%) | 2129 (57.14) |  |  |
| Residence |  |  |  | 0.14 (0.10, 0.17) | <0.001 |
| Urban | 10293 (60.71) | 7839 (59.26) | 2454 (65.86) |  |  |
| Rural | 6661 (39.29) | 5389 (40.74) | 1272 (34.14) |  |  |
| Household income in the previous year (RMB) |  |  |  | 0.11 (0.08, 0.15) | <0.001 |
| ≤10000 | 8332 (53.33) | 6368 (52.35) | 1964 (56.78) |  |  |
| 10000 to ≤50000 | 6561 (41.99) | 5180 (42.58) | 1381 (39.92) |  |  |
| >50000 | 731 (4.68) | 617 (5.07) | 114 (3.30) |  |  |
| Marital status, n (%) |  |  |  | 0.10 (0.07, 0.14) | <0.001 |
| Married or partnered | 11703 (69.03) | 9270 (70.08) | 2433 (65.30) |  |  |
| Other marital status | 5251 (30.97) | 3958 (29.92) | 1293 (34.70) |  |  |
| Education level (years), n (%) |  |  |  | 0.08 (0.05, 0.12) | <0.001 |
| No school | 10533 (62.15) | 8237 (62.29) | 2296 (61.64) |  |  |
| Primary school | 4669 (27.55) | 3565 (26.96) | 1104 (29.64) |  |  |
| High school and above | 1747 (10.31) | 1422 (10.75) | 325 (8.72) |  |  |
| Living state |  |  |  | 0.02 (-0.02, 0.05) | 0.409 |
| With family members or institution | 14392 (84.89) | 11245 (85.01) | 3147 (84.46) |  |  |
| Alone | 2562 (15.11) | 1983 (14.99) | 579 (15.54) |  |  |
| Smoking |  |  |  | 0.05 (0.01, 0.08) | 0.038 |
| No | 11353 (67.00) | 8881 (67.17) | 2472 (66.38) |  |  |
| Ever | 2627 (15.50) | 2076 (15.70) | 551 (14.80) |  |  |
| Current | 2966 (17.50) | 2265 (17.13) | 701 (18.82) |  |  |
| Drinking |  |  |  | 0.06 (0.02, 0.09) | 0.009 |
| No | 11726 (69.20) | 9166 (69.33) | 2560 (68.76) |  |  |
| Ever | 2285 (13.49) | 1820 (13.77) | 465 (12.49) |  |  |
| Current | 2933 (17.31) | 2235 (16.90) | 698 (18.75) |  |  |
| Exercise at present |  |  |  | 0.08 (0.05, 0.12) | <0.001 |
| No | 12308 (72.60) | 9712 (73.43) | 2596 (69.67) |  |  |
| Yes | 4645 (27.40) | 3515 (26.57) | 1130 (30.33) |  |  |
| BMI category, n (%) |  |  |  | 0.03 (-0.01, 0.06) | 0.386 |
| <18.5 | 5373 (32.53) | 4207 (32.74) | 1166 (31.79) |  |  |
| 18.5 to <24 | 8856 (53.61) | 6883 (53.56) | 1973 (53.79) |  |  |
| ≥24 | 2289 (13.86) | 1760 (13.70) | 529 (14.42) |  |  |
| Comorbidity |  |  |  | 0.02 (-0.01, 0.06) | 0.447 |
| None | 8178 (51.27) | 6352 (51.03) | 1826 (52.10) |  |  |
| 1 | 4928 (30.89) | 3853 (30.96) | 1075 (30.67) |  |  |
| 2 or above | 2846 (17.84) | 2242 (18.01) | 604 (17.23) |  |  |
| Cognitive impairment |  |  |  | 0.23 (0.19, 0.26) | <0.001 |
| No | 8778 (52.05) | 6525 (49.59) | 2253 (60.76) |  |  |
| Yes | 8088 (47.95) | 6633 (50.41) | 1455 (39.24) |  |  |
| Death |  |  |  | 0.04 (0.00, 0.08) | 0.031 |
| No | 3597 (26.95) | 2543 (26.44) | 1054 (28.29) |  |  |
| Yes | 9748 (73.05) | 7076 (73.56) | 2672 (71.71) |  |  |

| **Supplementary Table 4**. Characteristics of participants lost to follow-up versus participants in the final study. | | | | | |
| --- | --- | --- | --- | --- | --- |
| Characteristics | Total (%) | Excluded (%) | Included (%) | Standardize diff. | P-value |
| N (%) | 4902 | 1176 | 3726 |  |  |
| Age, years | 84.60 ± 10.77 | 82.84 ± 10.82 | 85.16 ± 10.70 | 0.22 (0.15, 0.28) | <0.001 |
| Gender |  |  |  | 0.00 (-0.06, 0.07) | 0.920 |
| Male | 2103 (42.90) | 506 (43.03) | 1597 (42.86) |  |  |
| Female | 2799 (57.10) | 670 (56.97) | 2129 (57.14) |  |  |
| Residence |  |  |  | 0.40 (0.34, 0.47) | <0.001 |
| Urban | 2997 (61.14) | 543 (46.17) | 2454 (65.86) |  |  |
| Rural | 1905 (38.86) | 633 (53.83) | 1272 (34.14) |  |  |
| Household income in the previous year (RMB) |  |  |  | 0.18 (0.11, 0.25) | <0.001 |
| ≤10000 | 2487 (54.90) | 523 (48.83) | 1964 (56.78) |  |  |
| 10000 to ≤50000 | 1869 (41.26) | 488 (45.56) | 1381 (39.92) |  |  |
| >50000 | 174 (3.84) | 60 (5.60) | 114 (3.30) |  |  |
| Marital status, n (%) |  |  |  | 0.17 (0.11, 0.24) | <0.001 |
| Married or partnered | 3102 (63.28) | 669 (56.89) | 2433 (65.30) |  |  |
| Other marital status | 1800 (36.72) | 507 (43.11) | 1293 (34.70) |  |  |
| Education level (years), n (%) |  |  |  | 0.25 (0.18, 0.32) | <0.001 |
| No school | 2915 (59.48) | 619 (52.64) | 2296 (61.64) |  |  |
| Primary school | 1468 (29.95) | 364 (30.95) | 1104 (29.64) |  |  |
| High school and above | 518 (10.57) | 193 (16.41) | 325 (8.72) |  |  |
| Living state |  |  |  | 0.04 (-0.03, 0.10) | 0.258 |
| With family members or institution | 4124 (84.13) | 977 (83.08) | 3147 (84.46) |  |  |
| Alone | 778 (15.87) | 199 (16.92) | 579 (15.54) |  |  |
| Smoking |  |  |  | 0.02 (-0.05, 0.09) | 0.829 |
| No | 3243 (66.18) | 771 (65.56) | 2472 (66.38) |  |  |
| Ever | 733 (14.96) | 182 (15.48) | 551 (14.80) |  |  |
| Current | 924 (18.86) | 223 (18.96) | 701 (18.82) |  |  |
| Drinking |  |  |  | 0.04 (-0.02, 0.11) | 0.459 |
| No | 3388 (69.16) | 828 (70.41) | 2560 (68.76) |  |  |
| Ever | 611 (12.47) | 146 (12.41) | 465 (12.49) |  |  |
| Current | 900 (18.37) | 202 (17.18) | 698 (18.75) |  |  |
| Exercise at present |  |  |  | 0.25 (0.19, 0.32) | <0.001 |
| No | 3274 (66.79) | 678 (57.65) | 2596 (69.67) |  |  |
| Yes | 1628 (33.21) | 498 (42.35) | 1130 (30.33) |  |  |
| BMI category, n (%) |  |  |  | 0.13 (0.06, 0.19) | <0.001 |
| <18.5 | 1474 (30.52) | 308 (26.51) | 1166 (31.79) |  |  |
| 18.5 to <24 | 2623 (54.31) | 650 (55.94) | 1973 (53.79) |  |  |
| ≥24 | 733 (15.18) | 204 (17.56) | 529 (14.42) |  |  |
| Comorbidity |  |  |  | 0.16 (0.09, 0.22) | <0.001 |
| None | 2321 (50.28) | 495 (44.55) | 1826 (52.10) |  |  |
| 1 | 1455 (31.52) | 380 (34.20) | 1075 (30.67) |  |  |
| 2 or above | 840 (18.20) | 236 (21.24) | 604 (17.23) |  |  |
| Cognitive impairment |  |  |  | 0.18 (0.12, 0.25) | <0.001 |
| No | 3067 (62.86) | 814 (69.51) | 2253 (60.76) |  |  |
| Yes | 1812 (37.14) | 357 (30.49) | 1455 (39.24) |  |  |

| Supplementary Table 5. Univariate analysis Cox regression analysis associated with all-cause mortality. | | | |
| --- | --- | --- | --- |
|  | all-cause mortality |  |  |
| N (%) | Statistics | HR (95% CI) | P-value |
| Age, years | 85.16 ± 10.70 | 1.06 (1.06, 1.07) | <0.0001 |
| Gender |  |  |  |
| Male | 1597 (42.86%) | 1.0 |  |
| Female | 2129 (57.14%) | 0.76 (0.70, 0.83) | <0.0001 |
| Residence |  |  |  |
| Urban | 2454 (65.86%) | 1.0 |  |
| Rural | 1272 (34.14%) | 1.05 (0.97, 1.14) | 0.2326 |
| household income in the previous year (RMB) |  |  |  |
| ≤10000 | 2131 (57.19%) | 1.0 |  |
| 10000 to ≤50000 | 1478 (39.67%) | 1.02 (0.94, 1.10) | 0.6621 |
| >50000 | 117 (3.14%) | 0.97 (0.78, 1.21) | 0.7775 |
| Marital status, n (%) |  |  |  |
| Married or partnered | 2433 (65.30%) | 1.0 |  |
| Other marital status | 1293 (34.70%) | 1.01 (0.92, 1.12) | 0.7775 |
| Education level (years), n (%) |  |  |  |
| No school (0 years) | 2296 (61.62%) | 1.0 |  |
| Primary school (1–6 years) | 1105 (29.66%) | 1.11 (1.01, 1.22) | 0.0232 |
| High school and above (at least 7 years) | 325 (8.72%) | 1.12 (0.96, 1.31) | 0.1393 |
| Living state |  |  |  |
| With family members or institution | 3147 (84.46%) | 1.0 |  |
| Alone | 579 (15.54%) | 0.93 (0.84, 1.03) | 0.1759 |
| Smoking |  |  |  |
| No | 2473 (66.37%) | 1.0 |  |
| Ever | 552 (14.81%) | 1.34 (1.20, 1.49) | <0.0001 |
| Current | 701 (18.81%) | 1.15 (1.03, 1.27) | 0.0101 |
| Drinking |  |  |  |
| No | 2563 (68.79%) | 1.0 |  |
| Ever | 465 (12.48%) | 1.16 (1.04, 1.31) | 0.0102 |
| Current | 698 (18.73%) | 1.09 (0.98, 1.21) | 0.1047 |
| Exercise at present |  |  |  |
| No | 2596 (69.67%) | 1.0 |  |
| Yes | 1130 (30.33%) | 0.97 (0.89, 1.05) | 0.4558 |
| BMI category, n (%) |  |  |  |
| <18.5 | 1187 (31.86%) | 1.0 |  |
| 18.5 to <24 | 2007 (53.86%) | 1.03 (0.95, 1.13) | 0.4226 |
| ≥24 | 532 (14.28%) | 0.92 (0.81, 1.05) | 0.1990 |
| Comorbidity |  |  |  |
| None | 1952 (52.39%) | 1.0 |  |
| 1 | 1138 (30.54%) | 1.07 (0.98, 1.17) | 0.1267 |
| 2 or above | 636 (17.07%) | 1.02 (0.91, 1.14) | 0.7419 |
| Cognitive impairment |  |  |  |
| No | 2261 (60.68%) | 1.0 |  |
| Yes | 1465 (39.32%) | 1.14 (1.05, 1.24) | 0.0020 |
| Number of teeth | 8.51 ± 9.98 | 0.99 (0.99, 1.00) | 0.0006 |

Notes: Adjusted for age (continuous)

Continuous variables were expressed as mean ± standard deviation (SD); Categorical variables are presented as counts (percentages).

| **Supplementary Table 6.** Sensitivity analysis by excluding participants (N = 453) with heart diseases or stroke at baseline. | | | | | | |
| --- | --- | --- | --- | --- | --- | --- |
|  | Model Ⅰ |  | Model Ⅱ |  | Model Ⅲ |  |
|  | HR (95% CI) | P-value | HR (95% CI) | P-value | HR (95% CI) | P-value |
| Tooth loss trajectories |  |  |  |  |  |  |
| Progressively mild loss | Ref |  | Ref |  | Ref |  |
| Progressively severe loss | 1.44 (1.13, 1.84) | 0.003 | 1.43 (1.12, 1.82) | 0.004 | 1.52 (1.18, 1.97) | 0.001 |
| Edentulism | 1.67 (1.35, 2.08) | <0.001 | 1.65 (1.32, 2.05) | <0.001 | 1.62 (1.23, 2.15) | <0.001 |
| P for trend | <0.0001 | | <0.0001 | | 0.003 | |

Abbreviations: BMI: Body Mass Index.

Model I, adjust for age (continuous) and gender.

Model II, adjust for age (continuous), gender, education level and marital status.

Model III, adjust for age (continuous), gender, education level, marital status smoking, drinking, exercise at present, BMI category, comorbidity, cognitive impairment, and baseline number of teeth.

Results of Multiple cox regression analysis were presented as Hazard Ratio (HRs) and 95% confidence intervals (CIs)
